# The Influence of Culture on the Lure of Choice, Mental Accounting, and Overconfidence

**DOI:** 10.3390/bs14030156

**Published:** 2024-02-21

**Authors:** Sebastian Hoffmann, Sajid Anwar

**Affiliations:** School of Business and Creative Industries, University of the Sunshine Coast, Sippy Downs, QLD 4556, Australia

**Keywords:** behavioral economics, behavioral finance, decision making, culture, lure of choice, mental accounting, overconfidence

## Abstract

In the contemporary globalized landscape characterized by international and intercultural decision-making processes, interconnected supply chains, and diverse customer relations, susceptibility to biases and heuristics poses a substantial threat to the efficiency of decision making. This research explores the relatively understudied influence of culture on individuals’ susceptibility to concepts derived from behavioral economics. Employing the Individual Cultural Values Scale (CVSCALE), we examine the impact of culture on the allure of choice, mental accounting, and overconfidence among 837 participants from Australia (AU), China (CN), Germany (GE), and the United States (US) through logistic regression analysis. At the individual level, discernible interactions between power–distance, allure of choice, and overconfidence are observed. On the national scale, power–distance (AU, US), uncertainty avoidance (US), and masculinity (CN) significantly impact the allure of choice, while overconfidence is influenced by power–distance (US) and masculinity (US). Our analysis shows that culture plays a pivotal role in shaping susceptibility to biases and heuristics, thereby influencing decision-making processes. The findings advocate for a culturally differentiated approach to behavioral economics, emphasizing the need to tailor strategies and interventions based on cultural nuances.

## 1. Introduction

In contrast to the neoclassic concept of Homo economicus, behavioral economics asserts that human rationality is influenced by cognitive biases and heuristics. Therefore, we are susceptible to irrational and inconsistent decision making [1]. Since the beginning of research on behavioral economics, researchers have refined and extended the body of knowledge in this field. Even established concepts, such as overconfidence, offer various starting points for further exploration [2]. In general, researchers find indications of the need for a culturally differentiated approach to behavioral economics [3]. 

There is a growing number of publications dealing with the exact impact of culture on finance and economics. For example, one study demonstrates a connection between countries with strong future-time reference languages and higher dividend payouts [4]. Other studies indicate that Chinese asset managers use intuition more than their Western counterparts [5] and confirm culturally differentiated reactions to colors which influence risk aversion [6]. An additional example is the findings on culture-based differences in risk perception and monetary aspiration [7].

Although recent studies support the case for a culturally differentiated approach towards behavioral economics, three limitations of past research persist. First, research regularly focuses on already well-studied biases and heuristics, such as overconfidence and loss aversion. Second, the quantity of cultural backgrounds directly compared is considerably narrow and often limited to two countries. Finally, and most significantly, culture is largely approached as a geographical matter and not analyzed in terms of its differentiable dimensions. Some studies connect research findings to available index values from Hofstede’s research and provide correlations between findings (for example, see [8,9,10]). Yet, only a minority of studies specifically collect data on cultural dimensions from participants [11], creating opportunity for deviations. 

The objective of this research is to refine the applicability of behavioral economics in different cultural backgrounds by addressing the following issues. First, this research includes the lure of choice as a less-studied heuristic to expand the theory. The lure of choice has not previously been analyzed across cultures to our best knowledge. Second, participants of the two largest economies in the world, the United States and China, are included in the analysis. Additionally, responses from Germany, a leading exporting nation, and Australia, a long-time frontrunner in the United Nations Human Development Index, are considered to provide a balanced selection of countries. Finally, the application of Hofstede’s dimensions addresses the need for a more specific approach towards culture [12]. By applying the 26 Individual Cultural Values Scale (CVSCALE) items, developed by [13], Hofstede’s cultural dimensions are measured at the individual level. Accordingly, immediate conclusions on the interaction between cultural dimensions and decision-making behavior can be drawn. Data on cultural dimensions is gained from the same participants of the behavioral economics experiments. 

Following the lead of cultural differences in behavioral economics, we hypothesize that not only culture in general but cultural dimensions have an influence on decision-making behavior associated with the lure of choice, mental accounting, and overconfidence. This would be in line with the previous findings regarding mental accounting by [8,14]. Likewise, the influence of cultural dimensions on overconfidence was previously reported by [8,15,16]. Our intentions are to refine findings on mental accounting and overconfidence by applying CVSCALE and expand the theory by adding the lure of choice to the cross-cultural comparison. 

The experimental setup combines and modifies previous approaches to measure the lure of choice, mental accounting, and overconfidence. In our study, proneness is measured as a weighted average based on a variety of questions and experiments for each concept. To quantify the lure of choice, we apply the floating lure design established by [17] and modify it to detect mental accounting tendencies. We also replicate and update one of the classic experiments by [18] to test for mental accounting in a more isolated manner. Overconfidence is measured based on its subcategories of overestimation, overplacement, and overprecision. 

Participants in Australia, China, Germany, and the United States are selected and approached via Qualtrics to ensure maximal randomization concerning the order of items and experiments presented, as well as assigned conditions. This allows us to control the sample for a matching distribution of age and gender in the four countries. For the analysis, ordinal logistic regression is applied in two steps. First, individual-level data (ILD), where answers from participants of all cultural backgrounds are considered simultaneously, are analyzed. Second, country-level data (CLD) are examined separately for each of the four cultural backgrounds. 

Our results at the individual level indicate a connection between power–distance and the lure of choice. More generally, contributors from China and Germany exhibit significantly lower levels of mental accounting and overconfidence compared to their US–American counterparts. Job and age are found to be significantly associated with mental accounting, as are job and education with overconfidence. At the country level, we detect interactions between power–distance and the lure of choice in the Australian and US–American sample. Additionally, uncertainty avoidance influences the lure of choice in the US–American sample as masculinity does in the Chinese sample. Power–distance and masculinity interact with overconfidence in the US–American sample. Education, age, and job determine behavior associated with the examined concepts of behavioral economics. These findings highlight the relevance of research on the applicability of behavioral economics concepts in different cultural contexts. 

### 1.1. The Lure of Choice

The lure of choice (LoC) is a contradiction to the concept of making rational choices between various options. In the sense of utility-maximizing Homo economicus or the so-called economic man, a choice would be a logical consequence of a rational decision-making process. This seems to be because the process of choosing is overlaid by the objective inclusion and computation of all the information available, which enables economic man to make utility-maximizing choices, which do not involve hesitation or further deliberation [19].

However, research suggests that choice can be lured, and, therefore, it is prone to external interference and influence. The underlying mechanism is rather simple. People prefer choices that enable them to make additional choices at a later stage of the decision-making process, although this does not influence the outcome because the initial options stay the same. This behavior is explained by the human tendency to keep options accessible in uncertain settings [17,20].

Based on their analysis, Ref. [17] conclude that the lure of choice is most likely caused by three factors. The first is the human tendency to keep searching for additional but largely useless information, leading to procrastination of commitment. This appears even if the additional information is not needed for the final decision [21,22]. Second, people tend to make decisions that keep them acting and anticipating rather than ending the process [23]. Finally, more options appear to be more auspicious than fewer options. This explanation seems especially applicable when the options are not assessed soundly in advance [24]. Based on previous research on the lure of choice, the following hypotheses have been developed:

**H1a.**  *(To be tested using individual-level data): Cultural traits, measured with cultural dimensions, have a statistically significant impact on decision making at the individual level, which is driven by the lure of choice.*

**H1b.**  *(To be tested using country-level data): Cultural traits, measured with cultural dimensions, have a statistically significant impact on decision making at the country level, which is driven by the lure of choice.*

### 1.2. Mental Accounting

Mental accounting (MA), a key concept of behavioral economics, was first introduced by Nobel laureate Richard Thaler. In 1980, Thaler describes the behavior of consumers as irrational, and he criticizes the absence of a model that covers actual consumers’ decisions. To fill this gap, he names the foundation of his research prospect theory, and he emphasizes concepts such as the sunk cost effect, which now is also referred to as the sunk cost fallacy, and the neglect of opportunity costs [25].

Thaler’s theory implies that humans have different explicit or implicit mental accounts which they use to assess and categorize financial issues. Different cognitive rules, which follow prospect theory, apply to different accounts. These findings contradict earlier beliefs about the perception and assessment of value by customers, which were largely based on rational determinations of utility [26].

Over the years, Thaler refined the concept of mental accounting and provided in-depth research into various aspects. One finding is that people group certain expenses in different accounts, for example regarding their car or residence, and they occasionally set up explicit or implicit budgets for each. Costs in the same account are less relevant and bothersome than costs of another account. Moreover, these accounts are balanced in different time frames. Some accounts are balanced daily, others are balances annually. Mental accounting can provide a perceived cost-benefit analysis either before or after financial decisions [27].

Other examples of mental accounting are the perceptions of gift cards versus credit cards [28] and rolling mental accounts which pass on rules to other assets [29]. Moreover, deliberate mental accounting in taxation is a possible indicator of a prosperous business [30]. Furthermore, recent research provides suggestions for a goal-based rational distribution of financial resources across mental accounts leading to a goal-based theory of utility rooted in mental accounting [31]. Based on previous research on mental accounting, the following hypotheses have been developed:

**H2a.**  *(To be tested using induvial-level data): Cultural traits, measured with cultural dimensions, have a statistically significant impact on decision making at the individual level, which is driven by mental accounting.*

**H2b.**  *(To be tested using country-level data): Cultural traits, measured with cultural dimensions, have a statistically significant impact on decision making at the country level, which is driven by mental accounting.*

### 1.3. Overconfidence

Overconfidence (OC) is long known as a central bias and catalyst in behavioral economics, and it is especially recognized for its adverse effects on decision making and judgments [32]. Overconfidence has three subcategories: overestimation, overplacement, and overprecision [33].

Overestimation refers to the human tendency to overrate actual individual achievements in terms of higher capability and smartness. Overplacement describes the predisposition to rank one’s own accomplishments higher than those of others and generally rank oneself higher than others. Overprecision refers to the observation that people are disproportionately sure about the accuracy of their judgments and assessments [34].

Examples of the effects of overconfidence are entrepreneurial and managerial failures [35,36,37], excessive trading [38,39,40], and inaccurate forecasts [41]. Additionally, stock market anomalies can be attributed to overconfidence as summarized by [42]. Overconfidence is also related to more serious issues, such as wars [43], gun use in general [44], and misconceptions about climate change [45]. Based on previous research on overconfidence, the following hypotheses have been developed:

**H3a.**  *(To be tested using induvial-level data): Cultural traits, measured with cultural dimensions, have a statistically significant impact on decision making at the individual level, which is driven by overconfidence.*

**H3b.**  *(To be tested using country-level data): Cultural traits, measured with cultural dimensions, have a statistically significant impact on decision making at the country level, which is driven by overconfidence.*

### 1.4. Culture

While several definitions of culture exist, in this paper we rely on Hofstede’s research and focus on culture as a system of collective values, which can differentiate members from one group from members of another group. This definition is grounded in anthropology, but it is also applicable to other fields like business [12].

Although different culture models have been used in the social sciences, Hofstede’s model is the one that is most applied [13,46]. Nevertheless, the model is not free from criticism, as is comprehensively outlined by [47,48,49]. Besides criticizing the underlying attitude of Hofstede and his method, some authors, such as [50], also disapprove of certain dimensions of culture deemed as insufficient. However, to ensure comparability and to construct this research on the decades-long foundation of research in culture, this research applies Hofstede’s model.

Power–distance (PDI) measures and evaluates the inequality in society regarding the distribution of power. Insights into how equality or inequality of power are perceived and handled are gained. Individualism versus collectivism (IND) depicts the contrast between self-invoked and community-orientated societies. This refers to the implicit or explicit cultural framework of this aspect. Masculinity versus femininity (MAS) describes more than behavior that varies by gender. It portrays the tendency of a society to show either assertiveness or modesty in different combinations and with varying emphasis. Uncertainty avoidance (UAI) refers to the degree to which insecurity is perceived as inconvenient. However, the future cannot be foreseen by anyone. Thus, the uncertainty avoidance index shows the cultural attitude towards ambiguity.

Besides the four original dimensions of culture, PDI, IDV, MAS, and UAI, long-term versus short-term orientation (LTO) was added to the theory. The observation that societies put different weights on past and present as on the future is described and measured with LTO. The latest cultural dimension added to Hofstede’s model is indulgence versus restraint (IVR). It was coined by [51] while analyzing data from the World Values Survey. IVR depicts the ambivalence between subjective well-being, or happiness, self-determined living, and the concern for leisure on the one hand and constraint because of limiting social norms on the other hand.

The development and application of Hofstede’s cultural model started with studies of IBM’s workforce. Hofstede’s aim, as in the latest Values Survey Module in 2013, is to compare different national samples which require relatively large sample sizes and lead to general conclusions on the national level [52]. Because of the focus of this research on individual decision making, the Individual Cultural Values Scale (CVSCALE) is applied. Developed by [13], this approach measures Hofstede’s cultural dimensions at the individual level using 26 items. CVSCALE is available and validated for the first five dimensions of culture, which are included in this research.

## 2. Methodology

First, ordinal logistic regression was used to analyze the individual-level data (ILD). Answers from participants from all four cultural backgrounds were considered simultaneously to detect how the independent variables (PDI, IND, MAS, UAI, LTO) influenced their susceptibility to lure of choice, mental accounting, and overconfidence. Second, an analysis of country-level data (CLD) was carried out separately for each of the four cultural backgrounds. Again, ordinal logistic regression was applied.

### 2.1. Experimental Design

**Figure 1:**
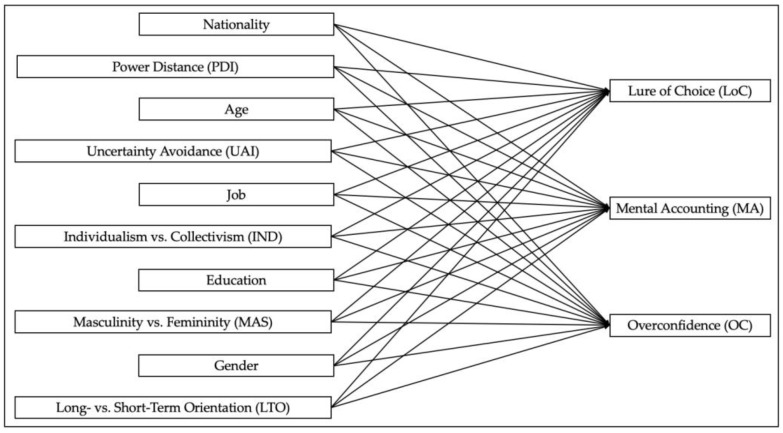
Independent and dependent variables.

Figure 1 provides an overview, including the mapping of the relationships, of the independent and dependent variables used in this research. 

Participants took a survey concerning their cultural background. It was based on Hofstede’s cultural dimensions and measured using the CVSCALE. Contributors provided their current nationality, nationality at birth, country of residence, age, gender, education, and job category. In study one, three questions regarding overconfidence were asked. In the first part of study two (the lure of choice), participants were randomly assigned to conditions in which decisions on a place to go out and eat and investing money had to be made. In the second part of study two, each participant had to solve two versions of a Monty Hall problem.

In study three (the lure of choice combined with mental accounting), participants were randomly assigned to a set of conditions. Participants had to answer two different questions on spending in study four (mental accounting). Taking the different cultural backgrounds into account, the survey and the studies were presented to participants in English, German, and Chinese, and they included adjusted amounts of money in the respective currencies: American Dollars (USD), Australian Dollars (AUD), Euros (EUR), and Renminbi (RMB). All three studies were conducted at the same time and with the same participants. The research was conducted double-blind. Qualtrics was used as a panel provider to ensure there were representative samples from the four countries in terms of age and gender. The minimum age to participate was 18 years.

### 2.2. Construction of Dependent Variables

The dependent variable overconfidence was calculated based on one question for each component of overconfidence: overplacement, overprecision, and overestimation. The queries concerning overplacement (general decision-making skills) and overestimation (likelihood of causing a car crash versus being in a car crash caused by someone else) were measured on seven-point Likert scales. For the item concerning overprecision (interval of which participants were 98% certain that the correct number of McDonald’s restaurants worldwide was included), the modulus between lower and higher thresholds was calculated and sorted from lowest to highest. As per the definition of overprecision, overly narrow confidence intervals are common when asked for estimates. A higher modus value shows a low level of overprecision. Then, the values derived from this item were divided into seven groups using SPSS. Based on the responses to the three items, a mean index value for overconfidence was calculated for each participant.

To test for the lure of choice, experiments established by [17,20] were altered and replicated to detect cultural differences. Experiments in study two applied the so-called floating lure design, in which participants were confronted with an isolated target (targetI) and another target (targetL) combined with a lure. This research design included pairing the lure with both targets for different groups of participants for randomization.

Participants had to choose between different pizza places to eat, based on information on taste, quality of service, and price. Two pizza places (lure and targetL) were on one side of a shopping mall and the third one (targetI) was in the opposite direction. Contributors were also asked to invest inherited money with a bank. Two banks were presented, one of which offered one investment (targetI) and the second offered two different investments (lure and targetL). Asset classes were fixed-rate bonds, equity funds, and savings accounts. To fulfill the predictions of the lure of choice, significantly more people were expected to choose the target paired with the lure and consequently prefer an option that enables a subsequent decision, even though the initial options remain unchanged. Participants were randomly assigned to conditions in which the restaurants were in opposite directions, and the names of the banks were changed.

In the second part of study two, participants were confronted with two conditions of a four-door Monty Hall problem: choose-a-door (CAD) and choose-a-choice (CAC). This version of the four-door Monty Hall problem was previously used by [17] and [20] in laboratory experiments in Great Britain. To emphasize the lure of choice, the three-door Monty Hall problem is expanded to a four-door version. This offers the opportunity to use different conditions for the experiment. The first is a condition in which participants can switch to a specific other door (CAD). In the second condition, participants must decide whether they want to choose another door and decide in favor of an additional choice as a first step without deciding on a specific door yet (CAC).

Concerning the experiment in which participants had to choose a place to eat and a bank to invest money with, responses were coded with the index value 1.0 when contributors chose the option that enabled an additional choice, showing the lure of choice. Regarding the Monty Hall experiments, participants who did not switch doors in the CAD condition but did switch them in the CAC condition were coded with the index value 1.0, indicating lure of choice behavior. Participants who switched doors in the CAD condition and decided on combined doors in the CAC condition were coded with the index value of 0.5. This indicated a tendency toward the lure of choice. Participants who did not switch doors in either condition and those who switched in the CAD but not in the CAC condition were coded with the index value 0.0. Based on the three experiments, a mean index value for the lure of choice was calculated.

Study three again used a version of the floating lure experiment designed by [17,20]. In that case, the experiments from the first part of study two (investing money with a bank) served as a basis. A second set of conditions was developed in which a mental accounting element was added in that the participant recently experienced a loss from an investment in equity funds.

To examine mental accounting behavior in a more isolated way, an altered version of a classic experiment originally developed by [18] was carried out. That experiment dealt with whether losing a ticket or losing the corresponding amount of cash led to different spending behavior when deciding whether to purchase another ticket or, respectively, to spend the amount of money anyway. During the original experiment, fewer people wanted to purchase another ticket after losing the first one. In contrast, participants frequently chose to spend the same amount on a ticket when the equivalent sum of cash was lost. This should not happen under a rational approach.

When there was a change in behavior regarding the loss of the movie ticket compared to the loss of cash, participants’ responses were coded with the index value of 1.0. If behavior did not change, the index value 0.0 was assigned. The same logic applied to the experiment about investing inherited money. When there was a change in behavior compared to the condition without a previous loss in a specific asset class, the index value 1.0 was assigned. When there was no change, the index value 0.0 was allocated. A mean value for mental accounting based on the three experiments was calculated and included in the analysis.

## 3. Results

All calculations, including constructing variables, calculating index values, descriptive statistics, and ordinal logistic regression were executed in SPSS. Participants’ age, gender, and nationality were asked among the first questions in the survey to fill the proposed quotas [53]. Unsuitable responses in this regard were eliminated during data collection. Responses were not considered for analysis when the duration of participation was below half of the median of the first 100 responses during the soft launch [54]. The respective minimum duration was set to 270 s, which equals four and a half minutes. In addition, flat-line responders were eliminated from the analysis. Flat-line responders were treated as such when the overall sum of answering values for the five cultural dimensions was below 82 or above 164, which equaled two standard deviations based on the data from the soft launch [55].

A total of 837 participants provided full data sets which were used for analysis, 208 from Australian, 205 from Chinese, 217 from German, and 207 from US–American contributors. A link to the survey and the experiments was sent out with the invitation to participate included in the matching default language which remained largely unchanged. Moreover, the country of residence mainly matched the participants’ nationality. Therefore, language and country of residence were subsequently eliminated from further analyses. The age of contributors ranged between 18 and 91 years with a mean of 45 years. Participants’ years of formal school education ranged between a minimum of 2 years and a maximum of 25 years, with a mean of 14 years.

Contributors provided their job as categorized by [52] as follows. Job 1: no paid job (includes full-time students)—13%; job 2: unskilled or semi-skilled manual worker—9%; job 3: generally trained office worker or secretary—14%; job 4: vocationally trained craftsperson, technician, IT-specialist, nurse, artist, or equivalent—17%; job 5: academically trained professional or equivalent (but not a manager of people)—19%; job 6: manager of one or more subordinates (non-managers)—18%; and job 7: manager of one or more managers—10%.

Cronbach’s alpha was calculated for the five cultural dimensions PDI (α = 0.84), UAI (α = 0.78), IND (α = 0.81), MAS (α = 0.77), and LTO (α = 0.73) after item five was deleted for this cultural dimension. The analysis of the influence of culture on the lure of choice, mental accounting, and overconfidence was first carried out on ILD. The complete dataset including all cultural backgrounds was analyzed simultaneously. Then, CLD were analyzed; the responses from the four different countries were analyzed separately. Table 1 provides descriptive statistics of the numerical variables. 

### 3.1. Individual-Level Data (ILD)

The findings are summarized in Table 2.

Concerning H1a, ordinal logistic regression on the lure of choice showed a significant negative effect of power–distance on the lure of choice (B = −0.155, *p* = 0.017). The finding shows that higher PDI values were associated with lower degrees of the lure of choice. An increase in the independent variable PDI by one led to a decrease in the odds for the lure of choice by the factor e^B^ = 0.856. Pseudo R^2^ was calculated at 0.032 (Nagelkerke).

Regarding H2a, there was no significant influence of the cultural dimensions on mental accounting. However, age was found to have a significant negative impact on this concept of behavioral economics (B = −0.190, *p* < 0.001). An increase in the variable age by one year led to a decrease in the odds for mental accounting by the factor e^B^ = 0.827. Thus, a higher age was significantly associated with lower mental accounting. Compared to the reference category, managers of one or more managers (job 7) and managers of subordinates (job 6) showed significantly lower mental accounting (B = −0.884, *p* = 0.004). Concerning job 6, the odds were calculated at e^B^ = 0.413 compared to the reference category (job 7) for mental accounting.

Participants from China (B = −0.701, *p* = 0.003) and Germany (B = −0.736, *p* = 0.002) showed significantly less mental accounting behavior compared to the reference category, the United States. The odds were calculated at e^B^ = 0.496 for the chance to exhibit mental accounting behavior as a German national compared to the reference category, the United States. Likewise, for the chance to exhibit mental accounting behavior as a Chinese national compared to the reference category, the United States, the odds were calculated at e^B^ = 0.479. Pseudo R^2^ was calculated at 0.089 (Nagelkerke).

Concerning H3a, power–distance was found to have a significant negative effect on overconfidence (B = −0.138, *p* = 0.026). Participants who reflected high index values for PDI showed significantly lower index values for overconfidence. An increase in one of the independent variable PDI led to a decrease in the odds for overconfidence by the factor e^B^ = 0.871. Education had a significant negative effect on overconfidence (B = −0.049, *p* = 0.013). Participants who had more years of formal education were less likely to exhibit overconfidence. An increase of one year of school education led to a decrease in the odds for overconfidence by the factor e^B^ = 0.952.

**Figure 2:**
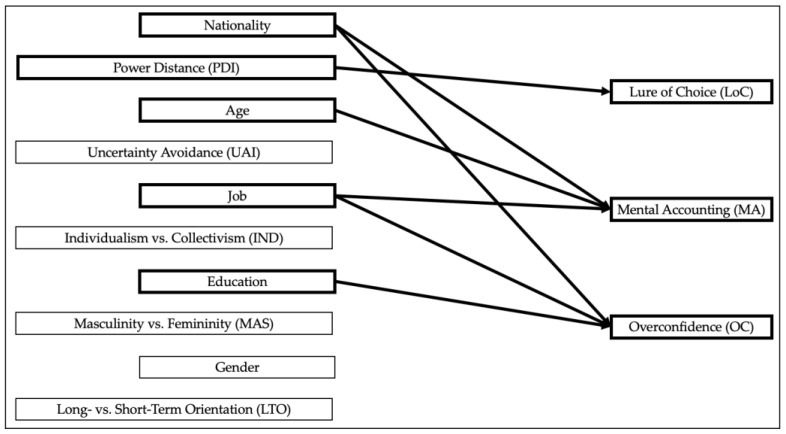
Visualization of significant results—individual-level data.

Regarding the jobs of participants, academically trained professionals (job 5) showed significantly lower overconfidence than managers of one or more managers (job 7) (B = −0.522, *p* = 0.029). Regarding job 5, the odds for overconfidence were calculated at e^B^ = 0.593 compared to the reference category (job 7). Participants from China (B = −0.590, *p* = 0.002) and Germany (B = −0.588, *p* = 0.002) exhibited significantly lower levels of overconfidence compared to participants from the United States. The odds were calculated at e^B^ = 0.554 for the chance to exhibit overconfidence as a Chinese national compared to the reference category, the United States. Likewise, for the chance to exhibit mental accounting as a German national compared to the reference category, the United States, the odds were calculated at e^B^ = 0.555. Pseudo R^2^ was calculated at 0.051 (Nagelkerke). The findings are visualized in Figure 2. 

### 3.2. Country-Level Data (CLD)

Table 3 provides an overview of the results from the four countries on the lure of choice.

Considering H1b, the Australian sample showed a significant negative finding on power–distance (B = −0.328, *p* = 0.037). Higher index values for PDI were associated with a weaker lure of choice. An increase of one of the independent variable PDI led to a decrease in the odds for the lure of choice by the factor e^B^ = 0.720. Pseudo R^2^ was calculated at 0.098 (Nagelkerke) for the Australian sample.

Considering the Chinese sample, masculinity was found to be a significantly negative factor (B = −0.381, *p* = 0.021). Higher masculinity was linked to lower index values for the lure of choice. An increase of one in MAS led to a decrease in the odds for the lure of choice by the factor e^B^ = 0.683. Generally trained workers (job 3) were found to have a significant positive impact on the lure of choice compared to managers of one or more managers (job 7) (B = 1.189, *p* = 0.041). Concerning job three, the odds were calculated at e^B^ = 3.284 for the lure of choice compared to the reference category (job 7). Pseudo R^2^ was calculated at 0.105 (Nagelkerke) for the Chinese sample.

Participants from Germany exhibited a significant negative interaction between education and the lure of choice (B = −0.113, *p* = 0.030). Contributors with more years of formal school education were less likely to exhibit behavior associated with the lure of choice. An additional year of schooling led to a decrease in the odds for the lure of choice by the factor e^B^ = 0.893. Pseudo R^2^ was calculated at 0.066 (Nagelkerke) for the German sample.

**Figure 3:**
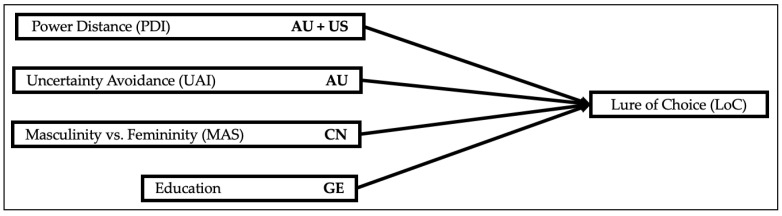
Visualization of significant results—country-level data: LoC.

In the American sample, power–distance (B = −0.364, *p* = 0.008) and uncertainty avoidance (B = −0.413, *p* = 0.040) showed significant negative effects on the lure of choice. Participants who scored high on PDI exhibited lower levels for the lure of choice. An increase of one for PDI led to a decrease in the odds for the lure of choice by the factor e^B^ = 0.695. Likewise, participants scored high on UAI index values. An increase of one in UAI led to a decrease in the odds for the lure of choice by the factor e^B^ = 0.662. Pseudo R^2^ was calculated at 0.065 (Nagelkerke) for the US–American sample. The findings are visualized in Figure 3. 

Table 4 provides an overview of the results from the four countries on mental accounting.

Regarding H2b, the Australian and Chinese samples did not show significant interactions concerning mental accounting behavior during the logistic regression. Pseudo R^2^ was calculated at 0.085 (Nagelkerke) for the Australian sample, and 0.138 (Nagelkerke) for the Chinese sample.

In the German sample, age was identified as a significant negative factor for mental accounting (B = −0.030, *p* = 0.010). Older participants showed fewer signs of mental accounting. An increase of one year led to a decrease in mental accounting by the factor e^B^ = 0.970. Pseudo R^2^ was calculated at 0.129 (Nagelkerke) for the German sample.

**Figure 4:**
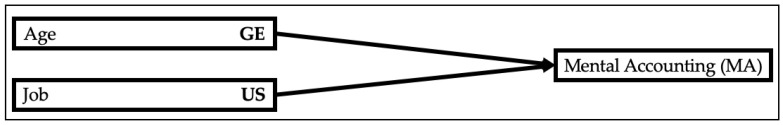
Visualization of significant results—country-level data: MA.

The analysis of data from the United States showed that participants who categorized themselves as managers of subordinates (job 6) were less likely to exhibit mental accounting behavior compared to managers of one or more managers (job 7) (B = −1.485, *p* = 0.014). The odds were calculated at e^B^ = 0.227 for mental accounting compared to the reference category (job 7). Pseudo R^2^ was calculated at 0.193 (Nagelkerke) for the US–American sample. The findings are visualized in Figure 4. 

Table 5 provides an overview of the results from the four countries on overconfidence.

Concerning H3b, the Australian and Chinese samples did not show significant results regarding overconfidence in the logistic regression. Pseudo R^2^ was 0.089 (Nagelkerke) for the Australian sample and 0.059 (Nagelkerke) for the Chinese sample.

The analysis of data from German participants resulted in one significant negative finding for the interaction between education and overconfidence (B = −0.104, *p* = 0.034). Participants who experienced more years of formal schooling were less likely to exhibit overconfidence. An additional year of school led to a decrease in overconfidence by the factor e^B^ = 0.901. Pseudo R^2^ was calculated at 0.068 (Nagelkerke) for the German sample.

**Figure 5:**
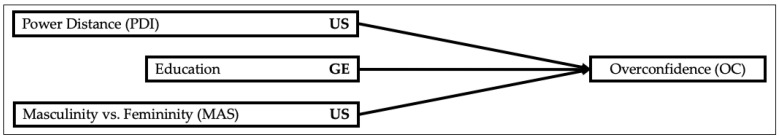
Visualization of significant results—country-level data: OC.

The logistic regression delivered two significant results regarding the sample from the United States. Power–distance showed a significant negative interaction with overconfidence (B = −0.310, *p* = 0.017). Participants who scored high on PDI showed lower values of overconfidence. An increase in PDI by one led to a decrease in overconfidence by the factor e^B^ = 0.733. Masculinity was found to have a significant positive relation to overconfidence (B = 0.277, *p* = 0.022). Contributors who scored high on masculinity also exhibited higher levels of overconfidence behavior. An increase of one for MAS led to an increase in the observable overconfidence by factor e^B^ = 1.319. Pseudo R^2^ was calculated at 0.086 (Nagelkerke) for the US–American sample. The findings are visualized in Figure 5. 

## 4. Discussion

Our analysis confirms that culture is indeed a factor in proneness to biases and heuristics and, therefore, in decision making. A culturally differentiated approach to behavioral economics appears advisable. Socio-demographic variables determine peoples’ behavior when challenged to make decisions and assessments. This was generally expected, given the circumstance that decision making is influenced by multiple factors and attributes [56]. The overall low values of Pseudo R^2^ in our analysis indicate the variety of influencing factors in decision making and that culture is only one of many influencing factors. 

At the individual level, our data specifically suggest that higher power–distance leads to a weaker lure of choice and less overconfidence. Chinese and German nationals exhibit significantly lower mental accounting and overconfidence compared to US–Americans. However, no further influence of cultural dimensions on the lure of choice, mental accounting, and overconfidence is detectable. At the same time, we find that age and job determine mental accounting, and age and education influence overconfidence. This leads to the assumption that socio-demographic factors can not be underestimated regarding their influence on concepts of behavioral economics. 

At the country level, we find various and heterogenous interactions between cultural dimensions, socio-demographic factors, and concepts of behavioral economics. Only the German sample does not show a connection between cultural dimensions and the three concepts of behavioral economics. Additionally, mental accounting is not determined by any cultural dimension. All but the Australian sample exhibit the influence of socio-demographic factors on decision making, underlining the differences between our samples. 

More research must be conducted to further refine the applicability of behavioral economics. This includes analyzing additional concepts of behavioral economics regarding culture as well as comparing additional cultural backgrounds, under continuous control for socio-demographic variables.

## Figures and Tables

**Table 1:** Descriptive statistics of numerical variables.

		PDI	UAI	IND	MAS	LTO	LoC	MA	OC	Age	Edu
Australia (n = 208)	M	2.77	5.70	4.44	3.36	5.62	0.52	0.18	4.64	48.41	13.86
	SD	1.22	0.84	0.95	1.29	0.77	0.24	0.26	0.90	17.14	3.83
	Min	1.00	2.60	1.00	1.00	3.80	0.00	0.00	2.33	18.00	2.00
	Max	6.60	7.00	7.00	6.75	7.00	1.00	1.00	6.67	80.00	25.00
China (n = 205)	M	3.48	5.61	5.13	4.65	5.85	0.50	0.13	4.35	41.30	14.63
	SD	1.38	0.75	0.87	1.07	0.67	0.25	0.25	0.88	13.53	2.87
	Min	1.00	3.20	2.50	1.50	3.80	0.00	0.00	1.67	18.00	2.00
	Max	6.60	7.00	7.00	6.75	7.00	1.00	1.00	7.00	70.00	25.00
Germany (n = 217)	M	2.94	5.18	4.92	3.74	5.36	0.47	3.74	4.39	43.09	12.09
	SD	1.24	0.85	0.92	1.29	0.88	0.25	1.29	0.93	15.58	2.71
	Min	1.00	3.20	2.17	1.00	1.00	0.00	0.00	2.00	18.00	5.00
	Max	6.00	7.00	7.00	7.00	7.00	1.00	1.00	7.00	76.00	24.00
United States (n = 207)	M	3.00	5.65	4.42	3.46	5.81	0.47	0.22	4.63	45.24	14.40
	SD	1.43	0.80	1.14	1.53	0.85	0.26	0.28	0.99	17.36	3.80
	Min	1.00	3.40	1.67	1.00	1.00	0.00	0.00	2.00	18.00	3.00
	Max	6.00	7.00	7.00	7.00	7.00	1.00	1.00	7.00	91.00	25.00
Total = (N = 837)	M	3.04	5.53	4.73	3.80	5.65	0.49	0.17	4.50	44.51	13.72
	SD	1.34	0.84	1.02	1.40	0.82	0.25	0.26	0.93	16.17	3.48
	Min	1.00	2.60	1.00	1.00	1.00	0.00	0.00	1.67	18.00	2.00
	Max	6.60	7.00	7.00	7.00	7.00	1.00	1.00	7.00	91.00	25.00

Note: Independent numerical variables are power–distance (PDI), uncertainty avoidance (UAI), individualism (IND), masculinity (MAS), long-term orientation (LTO), age, and education. Dependent numerical variables are the lure of choice (LoC), mental accounting (MA), and overconfidence (OC).

**Table 2:** Logistic regression analysis—individual-level data: LoC, MA, OC.

	B (LoC)	Wald (LoC)	*p* (LoC)	B (MA)	Wald (MA)	*p* (MA)	B (OC)	Wald (OC)	*p* (OC)	df
PDI	−0.155	5.671	0.017	0.081	1.031	0.310	−0.138	4.958	0.026	1
UAI	−0.063	0.399	0.528	−0.079	0.429	0.513	0.109	1.326	0.250	1
IND	0.118	2.416	0.120	0.069	0.562	0.453	0.068	0.897	0.344	1
MAS	0.022	0.123	0.725	−0.030	0.154	0.694	0.054	0.816	0.366	1
LTO	0.079	0.576	0.448	0.048	0.152	0.697	−0.093	0.889	0.346	1
Age	−0.006	1.870	0.171	−0.019	14.401	<0.001	0.004	0.860	0.354	1
Education	−0.014	0.456	0.500	−0.015	0.402	0.526	−0.049	6.188	0.013	1
Male	−0.230	0.044	0.833	−1.102	0.873	0.350	0.103	0.010	0.921	1
Female	−0.149	0.019	0.891	−0.930	0.627	0.429	−0.028	0.001	0.978	1
No Job/Student	0.205	0.531	0.466	0.136	0.185	0.667	−0.464	3.009	0.083	1
Unskilled Worker	−0.315	1.084	0.298	−0.608	2.861	0.091	−0.153	0.283	0.594	1
Generally Trained	0.137	0.244	0.622	−0.289	0.816	0.366	−0.160	0.368	0.544	1
Vocationally Trained	−0.131	0.250	0.617	−0.177	0.342	0.559	−0.234	0.874	0.350	1
Academically Trained	−0.166	0.439	0.507	−0.464	2.509	0.113	−0.522	4.787	0.029	1
Manager of Subordinates	0.207	0.675	0.411	−0.884	8.126	0.004	−0.124	0.269	0.604	1
Australia	0.264	2.035	0.154	−0.222	1.060	0.303	−0.032	0.034	0.854	1
China	0.126	0.398	0.528	−0.701	8.552	0.003	−0.590	9.642	0.002	1
Germany	−0.091	0.203	0.652	−0.736	9.354	0.002	−0.588	9.353	0.002	1

Note: The dependent variables are the odds ratio of the lure of choice, mental accounting, and overconfidence. Independent variables are power–distance (PDI), uncertainty avoidance (UAI), individualism (IND), masculinity (MAS), and long-term orientation (LTO). A Wald test (Wald) is carried out for each of the unstandardized regression coefficients (B), and the corresponding *p*-value (*p*) is determined. While B measures the influence of the independent variables in the regression model, odds ratio is used to interpret the respective coefficients.

**Table 3:** Logistic regression analysis—country-level data: LoC.

Lure of Choice	B (AU)	B (CN)	B (GE)	B (US)	Wald (AU)	Wald (CN)	Wald (GE)	Wald (US)	*p* (AU)	*p* (CN)	*p* (GE)	*p* (US)
Age	−0.006	−0.007	−0.005	−0.005	0.468	0.340	0.344	0.410	0.494	0.560	0.558	0.522
Education	−0.007	0.008	−0.113	0.033	0.035	0.024	4.707	0.000	0.852	0.877	0.030	0.999
PDI	−0.328	0.133	−0.100	−0.364	4.373	1.067	0.570	7.038	0.037	0.302	0.450	0.008
UAI	−0.004	−0.118	0.076	−0.413	0.000	0.172	0.153	4.236	0.983	0.679	0.695	0.040
IND	0.072	0.264	0.111	0.144	0.183	2.092	0.431	1.014	0.669	0.148	0.511	0.314
MAS	0.040	−0.381	0.136	0.182	0.086	5.313	1.323	2.065	0.769	0.021	0.250	0.151
LTO	−0.064	0.260	0.100	0.165	0.072	0.713	0.287	0.784	0.789	0.399	0.592	0.376
Male	−0.878	0.452	0.113	−0.635	0.194	2.495	0.181	0.211	0.659	0.114	0.671	0.646
Female	−0.437	−	−	−0.326	0.048	−	−	0.057	0.827	−	−	0.811
No Job/Student	0.430	0.776	−0.802	−0.081	0.517	1.074	−0.802	0.027	0.472	0.300	0.218	0.870
Unskilled Worker	−0.825	0.397	−0.535	−0.408	1.644	0.270	−0.535	0.614	0.200	0.603	0.446	0.433
Generally Trained	0.010	1.189	−0.710	0.041	0.000	4.168	−0.710	0.006	0.988	0.041	0.269	0.937
Vocationally Trained	−0.239	−0.019	−0.906	0.181	0.168	0.001	−0.906	0.098	0.682	0.970	0.141	0.754
Academically Trained	0.351	−0.321	−0.849	−0.288	0.387	0.428	−0.849	0.364	0.534	0.513	0.179	0.546
Manager of Subordinates	−0.034	0.639	−0.134	0.172	0.003	2.028	−0.134	0.139	0.956	0.154	0.837	0.709

Note: The dependent variable is the odds ratio of the lure of choice. Independent variables are power–distance (PDI), uncertainty avoidance (UAI), individualism (IND), masculinity (MAS), and long-term orientation (LTO). A Wald test (Wald) is carried out for each of the unstandardized regression coefficients (B), and the corresponding *p*-value (*p*) is determined. While B measures the influence of the independent variables in the regression model, odds ratio is used to interpret the respective coefficients.

**Table 4:** Logistic regression analysis—country-level data: MA.

Mental Accounting	B (AU)	B (CN)	B (GE)	B (US)	Wald (AU)	Wald (CN)	Wald (GE)	Wald (US)	*p* (AU)	*p* (CN)	*p* (GE)	*p* (US)
Age	−0.011	0.004	−0.030	−0.018	1.233	0.076	6.589	3.387	0.267	0.782	0.010	0.066
Education	0.019	−.060	0.065	−0.074	0.182	0.690	1.002	2.538	0.670	0.406	0.317	0.111
PDI	0.149	0.067	0.041	0.095	0.672	0.150	0.057	0.344	0.412	0.698	0.811	0.557
UAI	0.166	−0.120	0.011	−0.458	0.440	0.102	0.002	3.595	0.507	0.749	0.967	0.058
IND	−0.216	0.136	0.186	0.226	1.266	0.317	0.705	1.610	0.261	0.573	0.401	0.204
MAS	0.173	−0.416	0.134	−0.124	1.195	3.728	0.733	0.681	0.274	0.054	0.392	0.409
LTO	0.297	0.118	−0.181	0.334	1.142	0.086	0.604	1.922	0.285	0.769	0.437	0.166
Male	−2.878	−0.146	0.311	−1.833	1.753	0.149	0.838	1.396	0.185	0.700	0.360	0.237
Female	−2.613	−	−	−1.408	1.442	−	−	0.858	0.230	−	−	0.354
No Job/Student	−0.311	1.650	0.441	−0.159	0.216	3.619	0.350	0.081	0.642	0.057	0.554	0.776
Unskilled Worker	−1.044	−0.860	0.098	−0.708	1.867	0.630	0.014	1.336	0.172	0.427	0.906	0.248
Generally Trained	−0.200	0.393	−0.209	−0.520	0.083	0.316	0.074	0.802	0.773	0.574	0.786	0.371
Vocationally Trained	0.021	−0.179	−0.101	−0.293	0.001	0.074	0.020	0.211	0.973	0.785	0.889	0.646
Academically Trained	−0.453	−0.263	−0.447	−0.445	0.514	0.168	0.345	0.670	0.473	0.682	0.557	0.413
Manager of Subordinates	−0.800	−0.592	−0.405	−1.485	1.203	1.001	0.264	5.992	0.273	0.317	0.607	0.014

Note: The dependent variable is the odds ratio of mental accounting. Independent variables are power–distance (PDI), uncertainty avoidance (UAI), individualism (IND), masculinity (MAS), and long-term orientation (LTO). A Wald test (Wald) is carried out for each of the unstandardized regression coefficients (B), and the corresponding *p*-value (*p*) is determined. While B measures the influence of the independent variables in the regression model, odds ratio is used to interpret the respective coefficients.

**Table 5:** Logistic regression analysis—country-level data: OC.

Overconfidence	B (AU)	B (CN)	B (GE)	B (US)	Wald (AU)	Wald (CN)	Wald (GE)	Wald (US)	*p* (AU)	*p* (CN)	*p* (GE)	*p* (US)
Age	0.011	−0.010	0.002	0.009	2.129	0.874	0.050	1.434	0.145	0.350	0.823	0.231
Education	−0.022	−0.056	−0.104	−0.055	0.402	1.125	4.505	2.020	0.526	0.289	0.034	0.155
PDI	−0.121	−0.091	−0.056	−0.310	0.692	0.555	0.199	5.701	0.405	0.456	0.656	0.017
UAI	0.124	0.282	0.034	0.054	0.398	1.086	0.034	0.082	0.528	0.297	0.853	0.775
IND	−0.112	0.018	0.251	0.158	0.514	0.010	2.449	1.349	0.474	0.919	0.118	0.245
MAS	−0.023	0.071	−0.103	0.277	0.032	0.211	0.849	5.251	0.859	0.646	0.357	0.022
LTO	−0.093	−0.219	−0.047	−0.203	0.172	0.561	0.072	1.305	0.678	0.454	0.788	0.253
Male	−2.498	−0.037	0.054	1.236	1.823	0.018	0.047	0.874	0.177	0.892	0.829	0.350
Female	−2.631	−	−	1.056	2.012	−	−	0.658	0.156	−	−	0.417
No Job/Student	−0.574	−1.027	−0.544	0.024	1.045	2.073	0.791	0.003	0.307	0.150	0.374	0.960
Unskilled Worker	0.166	−0.849	0.012	−0.439	0.076	1.357	0.000	0.780	0.783	0.244	0.986	0.377
Generally Trained	−0.473	−0.618	0.154	0.077	0.645	1.270	0.065	0.025	0.422	0.260	0.799	0.874
Vocationally Trained	−0.193	−0.332	−0.351	−0.119	0.124	0.451	0.370	0.047	0.725	0.502	0.543	0.828
Academically Trained	−0.946	−0.677	−0.543	−0.030	3.156	2.095	0.837	0.004	0.076	0.148	0.360	0.948
Manager of Subordinates	0.380	−0.796	0.255	0.266	0.424	3.462	0.174	0.367	0.515	0.063	0.677	0.545

Note: The dependent variable is the odds ratio of overconfidence. Independent variables are power–distance (PDI), uncertainty avoidance (UAI), individualism (IND), masculinity (MAS), and long-term orientation (LTO). A Wald test (Wald) is carried out for each of the unstandardized regression coefficients (B), and the corresponding *p*-value (*p*) is determined. While B measures the influence of the independent variables in the regression model, odds ratio is used to interpret the respective coefficients.

## Data Availability

Data were obtained from consenting participants. As per the Research Project Information Sheet (RPIS) participants provided informed consent to the use of their data only by the specified research team.
